# A Case of Spontaneous Hydrophobia

**Published:** 1859-04

**Authors:** 


*A Case of Spontaneous Hydrophobia.* By Dr. Henrich.

F. K., thirty years of age, suffered on the twenty-ninth of May, 1857, of cephalalgia, which radiated from the forehead to the occiput, and of all the symptoms of a cold in the head. On the morning of the thirtieth, he complained of chills, and distressing horripilations. Dr. Henrich examined the patient attentively, without finding in the throat or elsewhere a single sign of disease. In the evening he was called in great haste to the patient, whom the found sitting in the bed,the face bathed in perspiration, pale and expressing terror; the eyes injected, brilliant, haggard; the voice hoarse, anxious, and broken. The patient complained of pain and constriction in the throat and chest, of intense thirst with impossibility to drink, and of dryness of the mouth. The respiratory movements were accelerated, superficial, and irregular; they became normal in the interval of the spasms, which followed each other rapidly; but when the throat became constricted, the patient seemed to suffocate, and carried the hand to the neck as if to remove an obstacle to respiration. The saliva flowed in great quantity from his mouth. Pulse ninety, and feeble. Pharynx a little reddened, and covered like the mouth with viscid mucus.

After earnest entreaties, Dr. Henrich finally succeeded in trying to overcome his violent horror of liquids; after having for a long time struggled against a convulsive contraction of the muscles of the forearm, he could finally bring a glass of water to his mouth, but hardly had the first few drops of the liquid touched his lips, when he was seized with a violent attack of suffocation. He threw his glass away with a gesture of despair, and taking refuge in the remotest part of the bed, cried out to take the water away; that he could not swallow; that he was suffocating.

In this condition he remained during the night. The impression of light or of a current of air exercised, however, no perceptible influence upon the spasms, and the vesicles of Morochetti were not found on the margin of the tongue. In spite of venesection, a blister on the chest, etc., all the symptoms were aggravated the next day; chloroform exasperated them; and they became less violent only for a few moments, after the patient had lost about a pound of blood through the wound made by the venesection, which had opened again; but soon they returned with greater intensity; tetanic convulsions and opisthotonos supervened, and the patient expired half an hour later. He had preserved the full power of his intellectual functions until tetanus came on.

On autopsy, a very slight swelling of the base of the tongue was discovered; the pharynx was in a healthy condition; some pulmonary hypostasis, and two hæmorragic suffusions in the mucous membrane of the stomach were found. All the other organs, the spinal marrow included, presented no alteration. The blood was black, liquid, and diffluent.

Dr. Henrich assured himself, by the most careful inquiries, that the patient had never been bitten by a dog, either mad or healthy, and that he did not believe himself at all attacked by hydrophobia. For three weeks previous, however, he was in low spirits, and without being otherwise sick, had a presentiment of his approaching death, as he said. He indulged, however, in excessive coïtus, (he was married and kept two mistresses,) and was troubled with grief and sorrow. To these two causes combined, the appearance of the terrible malady may be attributable.—(*Henke's Zeitschrift für Staatsarzneikunde*, 1858, p. 361.)

This case, which belongs to the third class of spontaneous hydrophobia of M. Chomel, (*Dict. de Méd.*, tome xv., 1837,) is, among all the published cases, one of the most characteristic. Cases of similar kind have been reported by MM. Ely, Burggreave, (*Gaz. des Hôpitaux*, 1854,) Lessmann, (*Preuss. Vereinszeitung*, 1854,) Bulley, (*Assoc. Med. Journ.*, 1854, Nov. 11,) and Putegnat, (*Journ. de Méd. de Bruxelles*, June, 1853.)—*Gazette Hebdomad.* 1858, 40,) from *The N. Amer. Medico-Chirurg. Review.*

